# A sensitive approach for simultaneous quantification of carbonyl and hydroxyl steroids using 96-well SPE plates based on stable isotope coded-derivatization-UPLC-MRM: method development and application†

**DOI:** 10.1039/c8ra01372a

**Published:** 2018-05-30

**Authors:** Chuanxin Liu, Xue Sheng, Yuming Wang, Jia Yin, Wei Huang, Yunshuang Fan, Yubo Li, Yanjun Zhang

**Affiliations:** Tianjin State Key Laboratory of Modern Chinese Medicine, School of Traditional Chinese Materia Medica, Tianjin University of Traditional Chinese Medicine 312 Anshan West Road Tianjin 300193 China yaowufenxi001@sina.com +86-22-59596221 +86-22-59596221; State Key Laboratory of Separation Membranes and Membrane Processes, Tianjin Polytechnic University Tianjin 300387 China; School of Environmental and Chemical Engineering, Tianjin Polytechnic University Tianjin 300387 China; Tianjin State Key Laboratory of Modern Chinese Medicine, Tianjin University of Traditional Chinese Medicine 312 Anshan West Road Tianjin 300193 China tianjin_tcm001@sina.com

## Abstract

Steroid hormones are crucial substances that mediate a wide range of vital physiological functions. Because of the important biological significance of steroids, this paper presents a new targeted metabolic method based on adding stable isotope tags to hydroxyl containing and carbonyl containing steroid hormones with two pairs of synthesized derivatization reagents: deuterium 4-(dimethylamino)-benzoic acid (D_4_-DMBA), and D_5_-Girard P (D_5_-GP) using of ultra performance liquid chromatography-multiple reaction monitoring (UPLC-MRM). Firstly, an Oasis PRiME hydrophilic-lipophilic balance (HLB) 96-well solid phase extraction plate was used to pretreat a number of biological samples simultaneously. Secondly, hydroxyl and carbonyl steroids were labeled using two pairs of synthetic reagents, namely DMBA and D_4_-DMBA, and GP and D_5_-GP, respectively. Thirdly, the mixed products were detected using UPLC-MRM and the mass spectroscopy conditions were optimized. Methodology development showed that the sensitivity was enhanced 1 to >500-fold. Finally, the new method was applied to analysis of urine samples of healthy males, females and rats. The results revealed that the method can be sensitive and reliable for simultaneous quantification of steroid hormones containing hydroxyl and carbonyl groups in 12 min in a single run. This method provided a powerful tool for studying the metabolic mechanism of steroids and contributed to the development of targeted metabolomics.

## Abstract 2

Steroid hormones are crucial substances that mediate a wide range of vital physiological functions.

## Graphical abstract

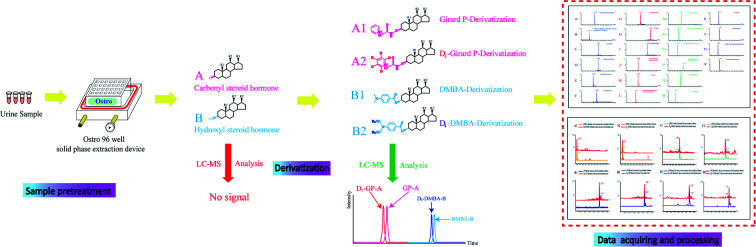

## Introduction

Steroid hormones are produced by the adrenal cortex, gonads and placenta, whereas cholesterol, which is in a class of four ring aliphatic hydrocarbons, is produced by an enzyme catalyzed by cytochrome P450. Steroid hormones have cyclopentane, phenanthrene, a mother nucleus with unique physiological and biochemical functions, which play an important role in the maintenance of life, regulation of sexual function, development of the body and so on.^1,2^ Many papers in the literature have reported that steroid hormones are associated with diseases, such as heart disease, high blood pressure, endocrine disorders, prostate cancer and so on.^3–6^ Considering the important biological implications of steroid hormones, it is necessary to build a sensitive and reliable method to determine multiple steroid hormones. Quantitatively measuring steroid hormones will also play a vital role in understanding disease progression and individual biochemical responses.^7^

The main methods reported for conventional analysis of steroid hormones include radio immunoassay (RIA),^8^ enzyme immunoassay (EIA),^9^ and gas chromatography-mass spectrometry (GC-MS).^10,11^ However, these methods experienced poor sensitivity, were labor intensive and lacked specificity. Chromatographic separation and identification using MS, enabled rapid qualitative analysis and accurate quantification of metabolites. Because of the high sensitivity of MS, lower levels of metabolites could be detected. Therefore, chromatography-mass spectrometry has an invaluable role in metabolomics research.^12,13^ Compared with the MS scan of non-target metabolomics, multiple reaction monitoring (MRM) in triple quadrupole-mass spectrometry (QQQ-MS) is increasingly used for work on targeted metabolism or the concentration analysis of homologues. Its high throughput and precise monitoring of complex matrices across a wide range of concentrations are its inherent advantages.^14^ Most researchers only focus on steroids that contain a kind of groups. However, the current study simultaneously quantified multiple classes of perssad, which improved the study of diseases related to steroid hormones. However, steroid hormones increased the difficulty of their detection because of their low ionization efficiency and low concentration characteristics.

Chemical derivatization is a commonly used sample pretreatment method in chromatographic analysis. It refers to the chemical reaction of a special chemical derivatization reagent with the analyte to change the physical and chemical properties of the analytes to be tested. Use of derivatization enhances the ability of analysis and the detection of steroids.^15^ Traditional quantitative analysis used to use a standard curve method which is generally used to measure the component *in vivo*. However the composition of the actual sample is different *in vivo*, and using the traditional methods will tend to bring some errors to the measurement. Some endogenous substances cannot be accurately quantified using a standard curve method. For complex mixtures, such as biological samples and environmental samples, the matrix effect can significantly change the ionization behavior of target analytes. Even for the same analyte, the ionization efficiency sometimes varies between two single runs.^16,17^ Stable isotopic derivatization is an alternative to introducing stable isotopes to the analyte, which can be used as a substitute for the internal standard. Before analysis, an analogue of the analyte was marked with stable isotopes, and were added as the internal standard to improve the accuracy by minimizing the matrix effect and ionization difference, so that the absolute/relative quantification will be more accurate when compared with the traditional standard curve method.

Based on the previously described research situation, a new method was proposed which is based on a 96-well solid-phase extraction (SPE) plate combined with a stable isotope labeling technique for simultaneous quantification of steroid hormones using means of UPLC-MRM. Firstly, an Oasis PRiME hydrophilic-lipophilic balance (HLB) 96-well SPE plate was used to pretreat all the biological samples simultaneously. Then, hydroxyl and carbonyl steroids were labeled using two pairs of synthetic reagents, namely by 4-(dimethylamino)-benzoic acid (DMBA) and deuterated-DMBA (D_4_-DMBA), Girard P (GP) and deuterated-GP (D_5_-GP), respectively. Thirdly, the mixed products were detected using UPLC-MRM and the mass spectroscopy conditions were optimized. Methodology development has shown that the method is sensitive and stable for the quantification of steroids. Finally, the new method was applied to urine samples from humans and rats. All the results revealed that the method is sensitive and reliable for simultaneous quantification of steroid hormones containing hydroxyl and carbonyl groups with a 12 min reaction in complex biological samples. The method provides a powerful tool for studying the metabolic mechanism of steroids and contributes to the development of targeted metabolomics.

## Experimental

### Reagents and materials

Estradiol (E_1_), estriol (E_2_) and cortisone were purchased from the Chinese National Institute for the Control of Pharmaceutical and Biological Products. 17α-Hydroxyprogesterone (17αOH-PROG), estrone (E_3_) and pregnanediol were purchased from TCI (Shanghai) Chemical Industry Development Co., Ltd. Progesterone (PROG) was purchased from Sigma-Aldrich (USA). Testosterone (TES) was purchased from the Tianjin Silan Technology Co., Ltd. Dehydroepiandrosterone (DHEA) was purchased from Adamas Regent. Pregnenolone (PREG) was purchased from Ark Pharm. Corticosterone, androsterone and 11-deoxycorticosterone were purchased from Dre (Germany). 5β-Pregnane-3,20-dione, 11β-hydroxyandrost-4-ene-3,17-dione, 19-hydroxyandrostenedione, 11β,17α,21-trihydroxy-5β-pregnane-3,20-dione, 2-methoxyestrone, cortol and tetrahydrocortisol were purchased from TRC (Canada). 17α-Hydroxypregnenolone (17αOH-PREG) and tetrahydrocorticosterone (THB) were purchased from ChromaDex (USA). The purity of all the standard products was greater than 98%.

HPLC-grade acetonitrile was purchased from Oceanpark (Gothenburg, Sweden). Distilled water was obtained from Watsons (Guangzhou, China). Ammonium acetate was purchased from Sigma-Aldrich (USA). The Ostro 96-well SPE plate was obtained from Waters Co., Ltd. (USA). Isoprenaline was obtained from Main Luck Pharmaceutical Inc. (China).

**Fig. 1:**
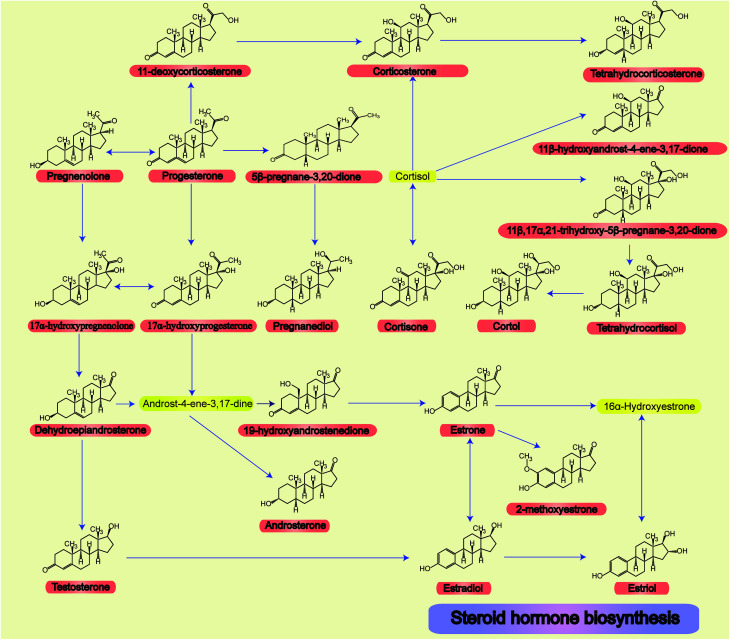
All 22 steroid hormone structures and their related metabolic pathways.

The relationships and structures of all steroid hormone related metabolic pathways are shown in Fig. 1.

### Synthesis of the derivatization reagents

**Fig. 2:**
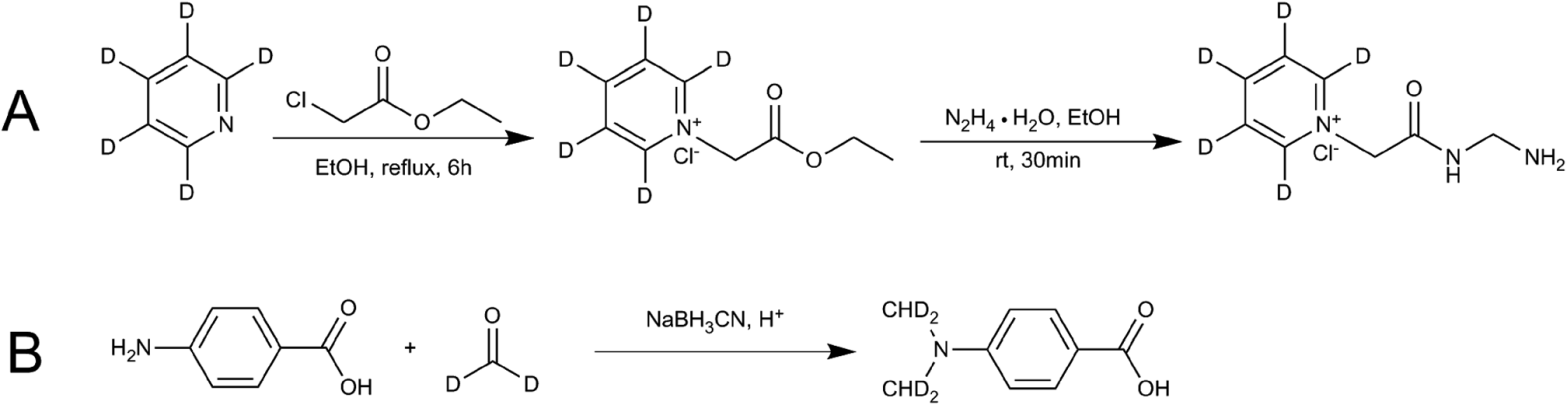
Reaction schemes for synthesis of the stable isotope-labeling reagent (A) D_5_-GP and (B) D_4_-DMBA.

Ethanol (EtOH) (12 mL), ethyl chloroacetate (6.13 g, 50 mmol) and D_5_-anhydrous pyridine (3.96 g, 50 mmol) were added to the flask to make a total of 100 mL. The mixture was heated and refluxed for 6 h. The resulting mixture was cooled to room temperature and then hydrazine hydrate (N_2_H_4_·H_2_O, 2.5 g, 50 mmol) was added. The product was allowed to precipitate for 30 min. The solid product was filtered out, washed with cold absolute EtOH, and then dried in a vacuum drying oven to obtain D_5_-GP. Finally, nuclear magnetic resonance spectroscopy (NMR), HPLC and MS were used to characterize the confirmation and purity of the product. Fig. 2A shows the synthetic scheme for D_5_-GP.

Glacial acetic acid (0.6 mL) was added dropwise to 4-carbamate (1.5 g) and dissolved in tetrahydrofuran (18 mL) and 1.8 mL of 20% D_2_-formaldehyde solution, followed by stirring. The solution was kept at room temperature for 1.5 h, then, 0.7 g of sodium cyanoborohydride (NaBH_3_CN) was added to the solution. After 30 min, 3.7 mL of D_2_-formaldehyde solution was added, followed by 1.5 g of NaBH_3_CN. The resulting solution was kept overnight at room temperature. The reaction liquid was poured into 50 mL of ice water and extracted twice with 100 mL of ethyl acetate. The ethyl acetate layer was dried using anhydrous magnesium sulfate and concentrated under pressure. The concentrated product was eluted using 200–300 mesh column chromatography and silica gel. The elution solvent of methanol/dichloromethane was used with an elution gradient of 0–10% to obtain the product. Finally, NMR, HPLC and liquid chromatography-mass spectrometry (LC-MS) were used to detect and characterize the samples. Fig. 2B shows the synthetic scheme for D_4_-4-(dimethylamino)benzoic acid (D_4_-DMBA).

### Preparation of a standard solution

A portion (1 mg) of each of the 22 standard steroid hormone products was weighed and placed in a 1 mL capacity bottle and dissolved in methanol to make a 1 mg mL^−1^ standard solution. Then the solution was diluted to the desired concentration in a volumetric flask.

### Animal treatment

The experimental animals were purchased from Sibei Fu Experimental Animal Science and Technology Co., Ltd. (Beijing, China), and the license number was SCXK (Jing) 2016-0011. The animal experiments were performed at the Institute of Radiation Medicine, Chinese Academy of Medical Sciences (Tianjin, China). Male Wistar rats weighing 200 ± 20 g were raised in an SPF level lab. The rats were housed under the following conditions: ambient temperature of 23 ± 2 °C and humidity of 35 ± 5%. This study was approved by the Animal Ethics Committee of Tianjin University of Traditional Chinese Medicine under permit number TCM-2012-078-F01. All experimental procedures were conducted in accordance with Chinese national legislation and local guidelines.

### Collection of biological samples

From healthy adult males (*n* = 6) and females (*n* = 6), midstream urine was collected in the morning. The urine samples were centrifuged at 760 × *g* for 15 min. The supernatant obtained was centrifuged at 1040 × *g* for 8 min. Then, the supernatant was extracted. Samples were handled gently to avoid turbidity from the precipitate. After the sample was combined with sodium azide (10 μL mL^−1^), the sample tube was placed upside down after adding the preservative so that the preservative was dispersed evenly. Finally, it was stored at −80 °C in preparation for further research.

The 12 hour urine of male Wister rats (*n* = 6) was collected, and the urine was centrifuged at 4 °C and 760 × *g* for 15 min. The supernatant was centrifuged at 1040 × *g* for 8 min. Then, the supernatant was extracted. After the supernatant was combined with sodium azide (10 μL mL^−1^), the sample tube was placed upside down. Finally, it was stored at −80 °C in preparation for further research.

### Use of 96-well SPE plates for pretreatment samples

Urine samples were treated to precipitate the proteins first. Acetonitrile (1500 μL) were added to 500 μL of urine to precipitate the protein. The resultant mixture was mixed ultrasonically in cold water for 10 min and vortex mixed for 1 min and then centrifuged at 14 360 × *g* for 15 min at 4 °C. The supernatants were loaded into each well of the Ostro 96-well SPE plate (Waters, USA) to remove the phospholipids. A vacuum was applied to the plate for 5–8 min. Then, the pretreatment samples were collected into centrifuge tubes, and nitrogen (N_2_) was used to dry and concentrate the samples before GP and DMBA derivatization.

### Derivatization

**Fig. 3:**
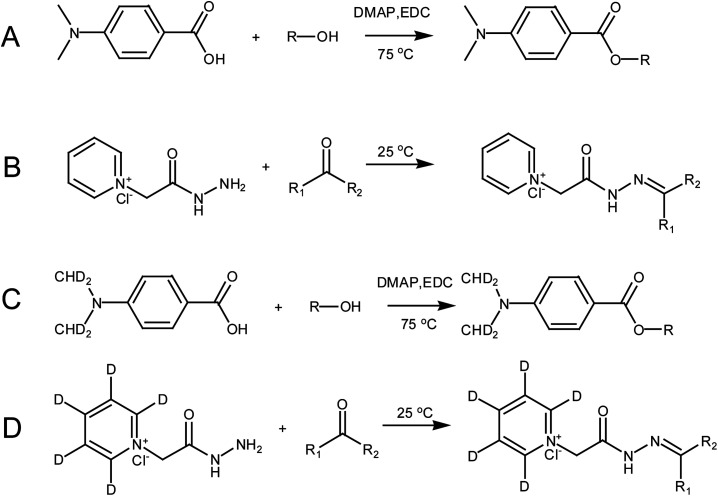
The derivatization scheme involved in this paper: (A) steroid hormones containing hydroxyl groups derivatized by DMBA; (B) steroid hormones containing carbonyl groups derivatized by GP; (C) steroid hormones containing hydroxyl groups derivatized by D_4_-DMBA; and (D) steroid hormones containing carbonyl groups derivatized by D_5_-GP.

D_4_-DMBA solution (100 μL, 2 mg mL^−1^), 4-dimethylaminopyridine solution (DMAP; 100 μL, 2 mg mL^−1^) and 1-ethyl-3-(3-dimethylaminopropyl) carbodiimide solution (EDC; 100 μL, 5 mg mL^−1^) were added successively to the nitrogen dried urine samples using methylene chloride as a solvent (Fig. 3C). After rigorous vortex mixing, the solution was transferred to a thick walled pressure bottle and sealed. The thick walled pressure bottle was kept at 75 °C for 5 h. After cooling the bottle, the sample was transferred to a centrifuge tube and centrifuged at 14 360 × *g* for 15 min to obtain the supernatant. Then, N_2_ was used to dry solution at 45 °C for 2 min. The residue was dissolved in 100 μL of 50% acetonitrile and the solution was then centrifuged at 14 360 × *g* for 15 min to give the supernatant. The supernatant was then ready for use. DMBA solution (100 μL, 2 mg mL^−1^, DMAP solution (100 μL, 2 mg mL^−1^) and EDC solution (100 μL, 5 mg mL^−1^) were added successively to the standard solution containing the 22 steroids dried by N_2_ (Fig. 3A). The rest of the methodology was the same as that used for urine samples.

A dry urine sample was derivatized with 150 μL of D_5_-GP (10 mM) reagent in a methanolic solution of 1% acetic acid (Fig. 3D). The sample was oscillated at room temperature for 120 min and dried under N_2_ at 45 °C. The residue was dissolved in 100 μL of 50% acetonitrile, which was centrifuged at 14 360 × *g* centrifugal force for 15 minutes to give the supernatant. The supernatant was then ready for use. The dry standard mixture containing the 22 steroids was derivatized with 150 μL GP (10 mM) reagent in a methanolic solution of 1% acetic acid (Fig. 3B). Other operations were the same as those performed with the urine samples.

Each of the two samples was derivatized with deuterated reagents, and the two deuterated derivatization products were mixed. The DMBA-standard or GP-standard (4 ng mL^−1^) was respectively added to D_4_-DMBA-urine samples and G_5_-GP-urine samples by 1 : 4 ratio. After mixing, the samples were analysed using UPLC-MRM.

### Apparatus and operation conditions

UPLC analysis was performed on a UPLC H-CLASS Xevo TQD system (Waters, USA). Sample aliquot (10 μL) were injected into an ACQUITY UPLC HSS T3 column (2.1 × 100 mm, 1.8 μm, Waters). The column temperature was set to 40 °C, and the flow rate was set to 0.4 mL min^−1^. The UPLC separation system included a binary solvent system with mobile phase A (0.1% ammonium acetate in water) and mobile phase B (acetonitrile). The gradient profiles were as follows: 0 min, (A: 100%); 0–1 min, (A: 100%); 1–2 min, (A: 100–70%); 2–5 min, (A: 70–55%); 5–6 min, (A: 55–30%); 6–9 min, (A: 30–0%); 9–10 min, (A: 0–0%); 10–11 min, (A: 0–100%); 11–12 min, (A: 100–100%). The H-CLASS Xevo TQD was equipped with electrospray ionization in positive and negative modes. In the positive ion multiple reaction monitoring (MRM) detection mode, the MS parameters were: drying gas temperature: 460 °C, capillary voltage: 1.5 kV, cone voltage: 50 V, atomizer: 7 bar, N_2_ solvent flow rate: 800 L h^−1^, conical airflow: 150 L h^−1^, impact gas flow: 0.14 mL min^−1^ and evaporation gas and auxiliary gas: N_2_. The scanning mode was used in the MRM and two sets of characteristic precursor ion/product ion pairs were selected, and their collision energy and declustering potential were optimized. A group of precursor ions/product ions with strong abundance was used for quantitative analysis. The entire MRM scan was divided into four segments to improve the operation efficiency and shorten the scanning cycle. The optimized parameters for the deuterated derivatization MS of the 22 endogenous steroids are shown in Fig. S1 (ESI†) and Table 1. The optimized parameters and detailed information for the MS of samples of 22 endogenous steroids without derivatization are shown in Table S1 (ESI†).

**Table 1:** Mass spectrometry information and conditions of deuterium derivatization substances

RT	Substance	Derivatization reagent	Precursor ion	Product ion	Dwell time (ms)	Collision energy (eV)	Cone voltage (V)
2.65	Cortisone	D_5_-GP	499.10	85.10	0.008	30	70
3.25	11β,17α,21-Trihydroxy-5β-pregnane-3,20-dione	D_5_-GP	503.20	85.00	0.008	30	74
3.20	17αOH-PREG	D_5_-GP	471.20	85.10	0.008	30	72
3.38	E_3_	D_5_-GP	409.10	157.00	0.008	30	62
3.58	THB	D_5_-GP	489.20	113.00	0.008	48	80
3.71	11β-Hydroxyandrost-4-ene-3,17-dione	D_5_-GP	441.20	357.23	0.008	24	60
3.75	TES	D_5_-GP	427.30	343.30	0.008	28	58
3.75	DHEA	D_5_-GP	427.20	80.0	0.008	26	58
4.04	11-Deoxycorticosterone	D_5_-GP	469.26	385.37	0.008	28	64
4.79	PREG	D_5_-GP	455.20	97.10	0.012	50	68
6.10	5β-Pregnane-3,20-dione	D_5_-GP	455.20	85.10	0.012	32	66
4.97	Androsterone	D_5_-GP	429.20	255.30	0.012	34	60
5.87	PROG	D_5_-GP	453.20	369.30	0.012	28	58
4.69	Cortol	D_4_-DMBA	520.20	152.10	0.012	24	20
9.49	E_2_	D_4_-DMBA	440.10	152.10	0.008	20	44
4.58	Tetrahydrocortisol	D_4_-DMBA	518.20	152.10	0.008	18	24
4.66	19-Hydroxyandrostenedione	D_4_-DMBA	454.10	152.10	0.008	22	30
5.25	17αOH-PROG	D_4_-DMBA	482.10	152.00	0.008	10	14
5.54	Corticosterone	D_4_-DMBA	498.20	152.10	0.008	26	52
6.53	E_1_	D_4_-DMBA	424.20	152.10	0.008	16	34
6.55	2-Methoxyestrone	D_4_-DMBA	452.20	152.10	0.008	16	36
8.46	Pregnanediol	D_4_-DMBA	472.20	170.10	0.008	36	48

### Data processing

Data acquisition, peak detection and peak alignment were performed using the Markerlynx function of the MassLynx software (version 4.1, Waters, USA). MS conditions were taken from the Intellistart (Waters, USA) using MS software optimization with repeated injections of a single component test solution. To obtain the optimal MS conditions for each analyte, the parameters were optimized, including the precursor ion, ion energy, and capillary voltage. Quantitative calculation of components use software Targetlynx (Waters, USA).

## Results and discussion

### Characterization and selection of derivatization reagents

Using suitable derivatization reagents is the key for detecting and quantifying steroids. Papers in the literatures report that reagents for the derivatization of hydroxyl groups were mainly picolinic acid,^18^ fusaric acid,^19^ 2-fluoro-1-methylpyridinium *p*-toluenesulfonate,^20,21^ isonicotinoyl azide,^22^ and DMBA.^23^ Reagents for the derivatization of carbonyl groups are mainly hydroxylamine,^24–27^ methoxylamine,^28^ Girard reagent T,^29^ GP,^30–34^ 2-hydrazino-1-methylpyridine,^35–37^ 2-hydrozinopyridine,^38,39^ 2-hydrazino-4-(trifluoromethyl)-pyrimidine^40^ and quaternary aminooxy.^41^

However, these derivatization reagents are complicated to use. They usually target only a class of hormones, which contains specific structures. DMBA has the advantage of increasing the ionization efficiency of steroid hormones by introducing the easily protonated tertiary amines. GP reagent has the advantage of stably introducing a permanently charged quaternary amine group to enhance the ionization efficiency. In this case, the synthesis of D_5_-GP and D_4_-DMBA could be used to provide an easily prepared isotope-labeled steroid hormone.

**Fig. 4:**
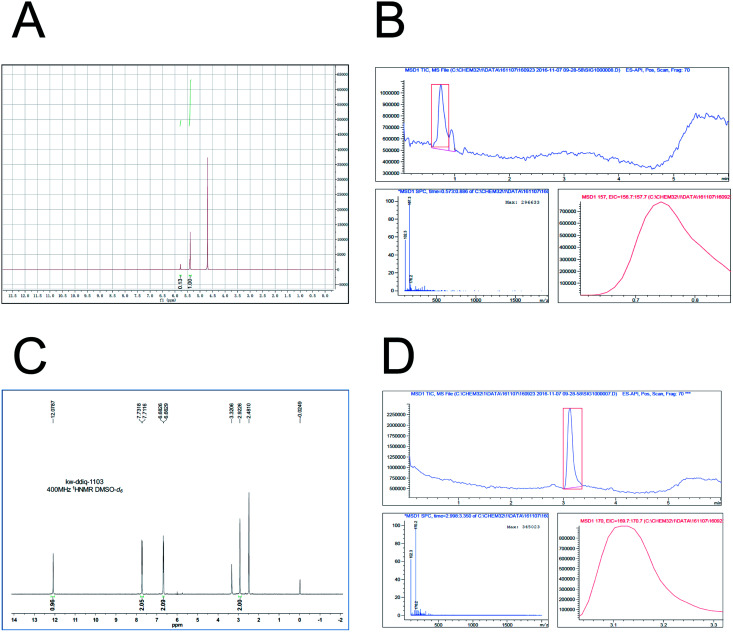
Both D_5_-Girard P and D_4_-DMBA chemical synthesis and characterization. (A) showed the successful synthesis of D_5_-Girard P by ^1^H-NMR (400 MHz-D_2_O); (B) showed the characterization of D_5_-Girard P by means of LC-MS (ES-API, Pos); (C) showed the successful synthesis of D_4_-DMBA by ^1^H-NMR (400 MHz-DMSO-*d*_6_); and (D) showed the characterization of D_4_-DMBA by means of LC-MS (ES-API, Pos).

Based on the previously described investigation, in this study D_5_-GP and D_4_-DMBA reagents were synthesized. D_5_-GP reagent (9 g) was obtained with a yield of 89%. For the D_4_-DMBA reagent (350 mg), the yield was 23.3% as determined using NMR and HPLC-MRM. The purity of synthetic D_5_-GP and D_4_-DMBA reached above 95% (Fig. 4)

### Optimization

In the derivatization of hydroxyl steroid hormones, methylene chloride was used as a solvent. These steroids were derivatized with DMBA (D_4_-DMBA) with DMAP and EDC as the catalysts. DMAP and DMBA (D_4_-DMBA) could be dissolved in dichloromethane, whereas EDC dissolved better in water. Therefore, in the early stage, EDC was dissolved in water to compare the MS response of the products after derivatization. The results are shown in Table S2 (ESI†). Of the 22 steroids, only eight substances could be detected, and their response value was lower than that of the products identified using dichloromethane as a solvent. These results were probably because of the density of water and dichloromethane. This led to a derivatization reaction with a response that was not complete at the interface.

In order to remove the endogenous interfering substances from the urine samples and to optimize the sample pretreatment method, a 96-well semi-automatic SPE method was used to handle a large number of samples.^42,43^ This method can remove phospholipid and proteins, which were the main components to interfere with the detection of endogenous substances.

Use of DMBA introduced tertiary amines, which were easily protonated, to label the hydroxyl groups. GP had the ability to introduce permanently charged quaternary amine groups to label the carbonyl groups. To identify the structures of the 22 steroids, hydroxyl steroids were derivatized using DMBA and carbonyl steroids were derivatized using GP. The steroids, which simultaneously contained carbonyl and hydroxyl groups, were difficult to quantify. In the early stage, two reagents were used for the derivatization. The results are compared and shown in Table S4 (ESI†). After optimization, the reagent with the best response value was used as the final derivatization reagent.

The UPLC-MRM conditions were optimized for the efficient separation of isomers and the enhancement of the mass response. For the mobile phase, the study compared formic acid water to ammonium acetate by the effect of the response value of steroid hormone detection. The results indicated that ammonium acetate can increase the ionization ratio of steroid hormones. The efficient separation of isomers was obtained using acetonitrile, water, and ammonium acetate as the mobile phase. Good MS results were dependent on the ions from the ammonium acetate. The optimized chromatographic peaks are presented in Fig. S1 (ESI†).

### Methodology development

Steroid hormone mixture solutions of 2, 5, 10, 20, 50, 100, 200, 500, 1000, 2000, 5000, 10 000, 20 000, 40 000 pg mL^−1^ were subjected to extraction, derivatization and UPLC-MRM analysis to evaluate the lowest limit of quantification (LLOQ) and linear range for the 22 derivatized steroid hormones. LLOQ was determined at the signal to noise ratio of 10. The results show that the corresponding non-deuterated steroid hormone LLOQ ranged from 5 pg mL^−1^ to 40 000 pg mL^−1^. Derivatization reagents were synthesized for labeling the steroid hormones to improve the sensitivity 1 to >500-fold with LLOQs ranging from 5 pg mL^−1^ to 5000 pg mL^−1^. Comparison of sensitivity of the UPLC-MRM detection of steroid hormones with and without derivatization by DMBA and GP are shown in Table S3 (ESI†). The results for the derivatized steroid hormones showed a good linear range. The regression coefficients ranged from 0.7944–0.9999 (Table 2).

**Table 2:** Lower limit of detection (LLOD), LLOQ, linear range, regression coefficient and stability for methodology development^a^

Steroid hormones	Derivatization reagent	LLOD (pg mL^−1^)	LLOQ (pg mL^−1^)	Linear range (pg mL^−1^)	Regression coefficient (*R*^2^)	Stability within 24 h RSD (%)
17αOH-PROG	DMBA	2	5	5–20 000	0.7944	1.3
E_1_	DMBA	5	10	10–20 000	0.9822	3.2
2-Methoxyestrone	DMBA	2	20	20–20 000	0.9881	6.2
Tetrahydrocortisol	DMBA	5	50	50–20 000	0.9942	3.1
Corticosterone	DMBA	20	50	50–20 000	0.9820	12
E_2_	DMBA	10	20	20–20 000	0.9941	15
Pregnandiol	DMBA	20	50	50–20 000	0.9911	3.4
19-Hydroxyandrostenedione	DMBA	50	200	100–40 000	0.9915	ND
Cortol	DMBA	100	200	200–40 000	0.9709	1.0
Androsterone	GP	<2	5	5–20 000	0.9999	2.5
11-Deoxycorticosterone	GP	2	5	5–20 000	0.9979	5.3
THB	GP	2	5	5–20 000	0.9991	8.8
5β-Pregnane-3,20-dione	GP	2	5	5–20 000	0.9984	1.4
PREG	GP	2	5	5–20 000	0.9981	ND
E_3_	GP	5	10	10–20 000	0.9997	7.2
TES	GP	2	10	10–20 000	0.9994	6.9
17αOH-PREG	GP	5	10	10–20 000	0.9993	1.5
DHEA	GP	5	20	20–20 000	0.9999	12
Cortisone	GP	20	50	50–40 000	0.9909	2.0
11β,17α,21-Trihydroxy-5β-pregnane-3,20-dione	GP	500	1000	1000–40 000	0.9967	ND
PROG	GP	2500	5000	5000–40 000	0.9986	ND

a ND: not detected.

Recoveries were investigated at low, medium and high levels. The different volumes of mixed steroid solutions were added to 500 μL of quality control (QC) samples. Then extraction, derivatization and UPLC-MRM analysis were performed to evaluate the recoveries. The results showed that the recovery rate complies with the requirements of methodological (Table 3).

**Table 3:** Recovery, inter-day precision and intra-day precision for methodology development^a^

Steroid hormones	Derivatization reagent	Recovery						Inter-day precision						Intra-day precision												
		LC (pg mL^−1^)	(%)	MC (pg mL^−1^)	(%)	HC (pg mL^−1^)	(%)	LC (pg mL^−^)	RSD (%)	MC (pg mL^−1^)	RSD (%)	HC (pg mL^−1^)	RSD (%)	LC (pg mL^−1^)	RSD (%)	MC (pg mL^−1^)	RSD (%)	HC (pg mL^−1^)	RSD (%)							
17αOH-PROG	DMBA	10	96.3	500	112	10 000	94.8	10	3.9	500	6.3	10 000	14	10	3.7	500	8.4	10 000	11							
E_1_	DMBA	50	117	500	81.2	10 000	81.2	50	2.2	500	1.3	10 000	9.0	50	10	500	1.2	10 000	11							
2-Methoxyestrone	DMBA	50	88.9	500	80.6	10 000	113	50	1.3	500	7.9	10 000	14	50	1.4	500	6.5	10 000	13							
Tetrahydrocortisol	DMBA	50	101	500	80.8	10 000	88.1	50	4.6	500	8.4	10 000	3.1	50	3.7	500	12	10 000	3.2							
Corticosterone	DMBA	50	101	500	79.2	10 000	107	50	1.8	500	11	10 000	12	50	2.7	500	7.4	10 000	11							
E_2_	DMBA	50	123	500	87.2	10 000	107	50	0.9	500	9.0	10 000	6.5	50	1.8	500	7.9	10 000	6.5							
Pregnandiol	DMBA	50	108	500	84.1	10 000	121	50	3.2	500	7.2	10 000	5.1	50	5.1	500	13	10 000	6.4							
19-Hydroxyandrostenedione	DMBA	500	ND	10 000	ND	40 000	ND	500	5.7	10 000	13	40 000	7.9	500	7.1	10 000	14	40 000	6.0							
Cortol	DMBA	500	91.2	10 000	95.0	40 000	70.2	500	1.3	10 000	3.1	40 000	2.7	500	1.7	10 000	15	40 000	2.8							
Androsterone	GP	10	80.1	500	85.2	10 000	102	10	3.9	500	2.3	10 000	4.6	10	5.7	500	2.0	10 000	3.5							
11-Deoxycorticosterone	GP	10	77.1	500	98.1	10 000	120	10	11.3	500	2.7	10 000	0.9	10	12	500	5.4	10 000	7.1							
THB	GP	10	96.7	500	82.6	10 000	91.0	10	4.9	500	7.7	10 000	4.0	10	4.2	500	6.7	10 000	4.1							
5β-Pregnane-3,20-dione	GP	10	101	500	83.2	10 000	93.5	10	1.3	500	3.6	10 000	3.1	10	1.5	500	4.1	10 000	3.5							
PREG	GP	10	82.0	500	77.5	10 000	91.4	10	3.2	500	1.0	10 000	1.5	10	2.9	500	7.5	10 000	3.0							
E_3_	GP	50	84.7	500	80.5	10 000	109	50	3.7	500	11	10 000	6.2	50	4.2	500	8.9	10 000	10							
TES	GP	50	91.5	500	102	10 000	89.7	50	6.1	500	1.2	10 000	4.7	50	2.5	500	1.3	10 000	2.8							
17αOH-PREG	GP	50	86.5	500	100	10 000	75.0	50	2.3	500	9.3	10 000	2.4	50	5.2	500	8.5	10 000	9.1							
DHEA	GP	50	99.4	500	105	10 000	96.7	50	1.9	500	1.8	10 000	3.4	50	1.6	500	2.5	10 000	2.1							
Cortisone	GP	50	84.1	500	94.6	40 000	104	50	8.5	500	4.7	40 000	1	50	5.6	500	9.2	40 000	1.1							
11β,17α,21-Trihydroxy-5β-pregnane-3,20-dione	GP	2000	88.2	10 000	84.8	40 000	85	2000	4.1	10 000	3.0	40 000	8.7	2000	4.3	10 000	6.6	40 000	12							
PROG	GP	5000	82.3	10 000	120	40 000	94	5000	1.3	10 000	1.5	40 000	1.9	5000	11	10 000	3.0	40 000	2.5							

a LC: low concentration. MC: medium concentration. HC: high concentration. ND: not detected.

QC samples (500 μL) were analyzed at 0, 4, 8, 12, 16, 20, 24 hours to test their stability within 24 hours. The relative standard deviation (RSD) value was calculated. The results showed that for the steroid hormones, the RSD were close to 15%, indicating that the method was stable (Table 2).

A mixed steroid solution was added to a 500 μL QC sample and then subjected to extraction, derivatization and UPLC-MRM analysis to evaluate the inter-batch and intra-batch precision (*n* = 6). The RSD values were also calculated. The results showed that the RSD range of the inter-batch accuracy was less than 15%. The RSD of intra-batch range accuracy was also less than 15%. These results showed that the precision complied with the requirements of the methodology (Table 3).

### Quantitative analysis of targeted metabolism

An analyte quantification approach based on the MRM with stable isotope labeled standard analytes provided absolute steroid hormone quantification values, which were based on the addition of known quantities of stable isotope labeled internal standards which were added to the urine samples. Quantification was achieved by comparing ion signals from the isotope labeled standards and the analytes. The biological samples labeled by deuterium derivatization reagents were mixed with a certain proportion of a steroid hormone standard labeled by non-deuterium derivatization reagents. Chromatograms showed two chromatographic peaks, which had different numbers of deuterated elements with *m*/*z* at the same retention times (RT). For example, the *m*/*z* value of the derivative products of D_5_-GP will be five more than that of the GP derivatives. The response value of the peak was proportional to the content of the product. So, accurate quantification of analytes was obtained using this technology, and the formula of quantification was:Area (internal standard)/area (analyte) = concentration (internal standard)/concentration (analyte)

**Fig. 5:**
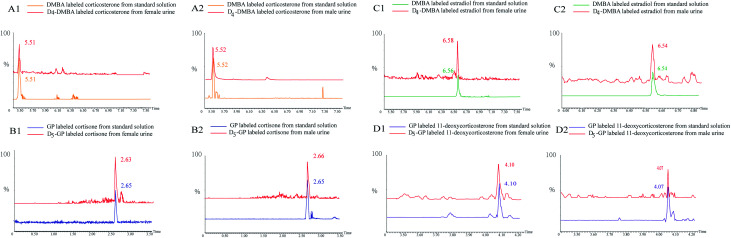
Peaks of quantification based on D/H stable isotope DMBA and GP labeling: extracted ion chromatogram of heavy and light labeled substances.

In Fig. 5, several substances are listed which include the peaks of the urine samples and inter standard solutions. The ion pairs of the D_4_-DMBA and the DMBA derivatized steroid hormone were found in a UPLC-MRM run. DMBA-corticosterone was determined to have an *m*/*z* of 494.2230, whereas D_4_-DMBA-corticosterone showed an *m*/*z* of 498.2139. The difference in *m*/*z* values showed that four hydrogen atoms had been replaced by four deuterium atoms. The D_4_-DMBA-analyte mixed with the DMBA-standard as internal standards, can be used to provide accurate and convenient quantifications of steroid hormones. The traditional standard curve method was more suitable for testing of samples *in vitro*. For substances *in vivo*, the standard curve method had a large error because of different compositions.^44,45^ Compared with the standard curve method, the isotope labeling method had more advantages in reducing the matrix effect and retaining stability.

### Application of the method for quantification

In this study, urine samples from six healthy females and males were subjected to steroid hormone quantification. The levels of steroid hormones in the urine samples of six rats were also quantified. GP derived steroid hormones and DMBA derived steroid hormones were detected in non-hydrolytic urine samples using UPLC-MRM analysis. The levels of these steroids were definitively quantified. The quantitative results are shown in Table 4. As expected, urine samples from males had higher levels of androgens. For example, TES in male urine samples was 3.8 times higher than in female samples and androsterone in male urine samples was 2.4 times higher than in female samples. More estrogen was found in female sample than in male samples. For example, the content of E_1_ in female urine samples was 4.4 times higher than in male samples, and E_3_ in female samples was 3.7 times higher than in male samples. The results obtained were consistent with those found in the literature.^45^ The urine samples from healthy males had higher hormone levels than the urine samples from male rats. For example, DHEA from healthy males was 3.2 times higher than in the samples from male rats. Androsterone from healthy males was 8.9 times higher than in the samples from male rats. Some substances like cortisone did not be detected because activity of 17-hydroxylase is so low in rodents that cortisol and cortisol can not be synthesized. Also, some progesterone steroids will be detected only in pregnant females. All these results are consistent with actual situations.

**Table 4:** Steroid levels in urine of healthy adult males, females and rats^a^

Substance	Derivatization reagent	Average content of urine samples in female (pg mL^−1^)		Average content of urine samples in male (pg mL^−1^)		Average content of urine samples in male rats (pg mL^−1^)
		This study	Reference ^45^	This study	Reference ^45^	
Cortol	DMBA	1326	—	13 340	—	ND
E_2_	DMBA	216.1	366	46.14	34	25.22
Tetrahydrocortisol	DMBA	884.1	902	3166	2588	2812
Corticosterone	DMBA	122.8	466	396.4	1013	ND
E_1_	DMBA	52.89	41	12.15	20	14.54
2-Methoxyestrone	DMBA	74.95	—	37.88	—	84.00
Cortisone	GP	38 766	—	23 479	—	ND
17αOH-PREG	GP	3343	508	ND	ND	ND
E_3_	GP	188.1	877	51.04	242	ND
THB	GP	228.3	—	244.9	—	ND
TES	GP	4234	16 626	15 988	17 884	16.68
DHEA	GP	1989	5329	5089	6851	1602
11-Deoxycorticosterone	GP	43.41	ND	34.13	35	ND
PREG	GP	75.74	ND	ND	ND	ND
5β-Pregnane-3,20-dione	GP	262.8	—	ND	—	ND
Androsterone	GP	1145	3529	2773	5999	312.9

a ND: not detected. —: relevant data not found.

## Conclusion

There is increasing evidence that steroid hormones play an important role in the development and progression of various diseases and quantitative metabolomics. However, few methods were established for detecting and quantifying steroid hormones comprehensively and high-sensitively. In this study, a stable isotope labeled derivatization method, using HPLC-MRM, was developed for targeted analysis of steroid hormones, which contain both carbonyl and hydroxyl groups. DMBA, D_4_-DMBA, GP, and D_5_-GP were synthesized as novel derivatization reagents for labeling steroid hormones and use of these improved the sensitivity from 1 to >500-fold with LLOQ ranging from 5 pg mL^−1^ to 5000 pg mL^−1^. The method had good inter-batch accuracy, intra-batch accuracy and stability over 24 h, and almost all the RSD were lower than 15%. The results showed that accurate relative and absolute quantification can be achieved using stable isotope labeling strategies. In addition, the method was successfully applied to the determination of healthy urine steroid hormones in males and females and in male rats. This method of derivation of steroid hormones based on stable isotope labeling techniques using the UPLC-MRM analysis platform can be used in steroid hormone metabolomics studies. Finally, the method established in this study not only accurately detected and quantified multiple steroid hormones in 12 min, but also provides guaranteed detection and quantification of steroid hormones in complex biological samples.

## Conflicts of interest

The authors declared no competing financial interest and no conflicts.

## Supplementary Material

RA-008-C8RA01372A-s001
