# Supplementary material for: A sensitive approach for simultaneous quantification of carbonyl and hydroxyl steroids using 96-well SPE plates based on stable isotope coded-derivatization-UPLC-MRM: method development and application
Source: RSC Adv. 2018 May 30;8(35):19713–23. doi: 10.1039/c8ra01372a (PMC9080693; doi:10.1039/c8ra01372a)
Supplement: RA-008-C8RA01372A-s001 [file RA-008-C8RA01372A-s001.pdf]

Electronic Supplementary Material (ESI) for RSC Advances.
This journal is © The Royal Society of Chemistry 2018

Electronic Supplementary Information

A sensitive approach for simultaneous quantification of carbonyl and hydroxyl steroid using 96-well SPE plates based on stable isotope coded-derivatization-UPLC-MRM: method development and application

Chuanxin Liu[&a], Xue Sheng[&a], Yuming Wang[&a], Jia Yin[a], Wei Huang[a], Yunshuang Fan[b,c], Yubo Li[*a] and Yanjun Zhang[*d]

[a]*Tianjin State Key Laboratory of Modern Chinese Medicine, School of Traditional Chinese Materia Medica, Tianjin University of Traditional Chinese Medicine, 312 Anshan west Road, Tianjin 300193, China.*

[b]*State Key Laboratory of Separation Membranes and Membrane Processes, Tianjin Polytechnic University, Tianjin 300387, China.*

[c]*School of Environmental and Chemical Engineering, Tianjin Polytechnic University, Tianjin 300387, China.*

[d]*Tianjin State Key Laboratory of Modern Chinese Medicine, Tianjin University of Traditional Chinese Medicine, 312 Anshan west Road, Tianjin 300193, China.*

[&]**Co-first authors:** Chuanxin Liu, Xue Sheng and Yuming Wang

[*]**Author for correspondence:**

Prof. Yubo Li

Tianjin State Key Laboratory of Modern Chinese Medicine, School of Traditional Chinese Materia Medica, Tianjin University of Traditional Chinese Medicine, 312 Anshan west Road, Tianjin 300193, China. E-mail: yaowufenxi001@sina.com.

Prof. Yanjun Zhang

Tianjin State Key Laboratory of Modern Chinese Medicine, Tianjin University of Traditional Chinese Medicine, 312 Anshan west Road, Tianjin 300193, China. E-mail: tianjin_tcm001@sina.com.

Tel and Fax number: +86-22-59596221.

Experimental

Chromatographic and mass spectrometric conditions of without derivatization

UPLC analysis was performed on a UPLC H-CLASS Xevo TQ-D system (Waters, USA). Urine samples (10 μL) were injected into an ACQUITY UPLC HSS T3 column (2.1×100 mm, 1.8 μm, Waters). The column temperature was set to 40°C, and the flow rate was set to 0.4 mL/min. The UPLC separation system includes a binary solvent system with mobile phase A (0.1% ammonium acetate in water) and mobile phase B (acetonitrile). The gradient profiles for the urine samples were as follows: 0 min, A: 99%; 0–1.5 min, A: 33%; 1.5-10min, A: 0%; 10-11min, A: 99%. The H-CLASS/Xevo TQ-D was equipped with electrospray ionization in the positive and negative modes. In the positive ion MRM detection mode, the MS parameters were as follows: drying gas temperature, 460°C; capillary voltage, 1.5 kV; cone voltage, 50 V; atomizer, 7 bar; nitrogen solvent flow rate, 800 L h^{-1}; conical airflow, 150 L h^{-1}; impact gas flow: 0.14 mL/min; and evaporation gas and auxiliary gas: nitrogen. The scanning mode was used for multiple reaction monitoring (MRM); two sets of characteristic precursor ion/product ion pairs were selected, and their collision energy and declustering potential were optimized. A group of precursor ion/product ion with strong abundance was used for quantitative analysis (Table S1).

Figure

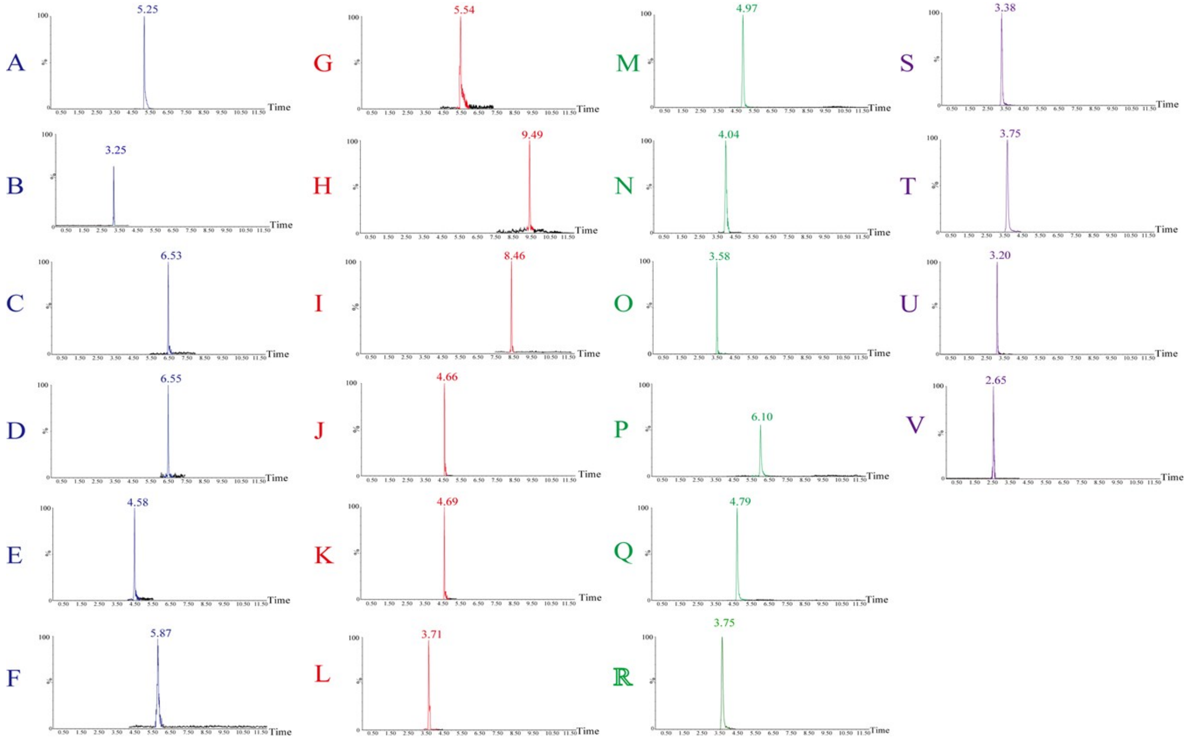

Fig S1. Chromatographic information for 22 steroid hormone standards. (A) 17αOH-PROG (RT:5.25); (B)11β,17α,21-trihydroxy-5β-pregnane-3,20dione (RT:3.25); (C) E_1 (RT:6.53);(D) 2-Methoxyestrone (RT:6.55); (E) Tetrahydrocortisol (RT:4.58); (F) PROG (RT:5.87); (G) Corticosterone (RT:8.52); (H) E_2 (RT:9.49); (I) Pregnandiol (RT:8.46); (J) 19-Hydroxyandrostenedione (RT:4.66); K) Cortol (RT:4.69); (L)11β-Hydroxyandrost-4-ene-3,17-dione (RT:3.71) (M) Androsterone (RT:4.97) ; (N)11-Deoxycorticosterone (RT:4.04); (O) THB (RT:3.58); (P) 5β-Pregnane-3,20-dione (RT:6.10);(Q) PREG (RT:4.79); (R) DHEA (RT:3.75); (S) E_3 (RT:3.38); (T) TES (RT:3.75); (U) 17αOH-PREG (RT:3.20); (V). Cortisone (RT:2.65).

Table S1. Mass spectrometry information and conditions of non-derivatization substances

RT	Substance	Precursor ion	Product ion	Dwell time (ms)	Collision energy（eV）	Cone voltage
3.83	Cortisone	361.2	163.3	0.009	24	50
4.04	11β,17α,21-Trihydroxy-5β-pregnane-3,20-dione	365.1	333.1	0.009	6	54
4.49	E_3	271.3	133.2	0.009	38	22
4.64	THB	351.3	315.3	0.009	6	30
4.8	11β-Hydroxyandrost -4-ene-3,17-dione	303.2	105.2	0.009	40	42
4.85	TES	289.2	97.0	0.009	22	44
4.86	DHEA	289.2	253.3	0.009	12	28
5.11	11-Deoxycorticosterone	331.2	109.1	0.009	28	40
5.73	PREG	317.2	285.2	0.009	4	30
5.73	5β-Pregnane -3,20-dione	317.3	43.1	0.009	34	34
6.68	PROG	315.2	97.1	0.009	26	42
8.59	Cortol	368.3	60.1	0.009	38	64
7.98	Tetrahydrocortisol	367.0	335.1	0.009	6	54
8.08	19-Hydroxyandro stenedione	303.2	91.1	0.009	44	44
8.54	17αOH-PROG	331.3	97.1	0.009	22	40
8.52	Corticosterone	347.3	121.1	0.009	22	62
9.54	E_1	272.9	243.5	0.009	8	86
9.57	2-Methoxyestrone	301.2	186.6	0.009	22	46

10.87	Pregnanediol	321.3	43.2	0.009	46	50

Table S2. The study on the conditions of derivation

Substance	Mass spectrometric response to EDC derivatization with water	Mass spectrometric response to EDC derivatization with dichloromethane
Cortol	4.10 e^6	4.12 e^6
E_2	8.80 e^5	1.20 e^6
Tetrahydrocortisol	4.16 e^5	4.54 e^5
19-Hydroxyandrostenedione	6.28 e^5	6.9 e^5
17αOH-PROG	7.40 e^6	7.12 e^6
Corticosterone	5.28 e^5	5.9 e^5
E_1	2.83 e^5	2.34 e^5
2-Methoxyestrone	5.73 e^5	5.24 e^5
Pregnanediol	5.60 e^5	5.79 e^5

Table S3. Comparison of sensitivity of UPLC-MS/MS detection of steroid hormone without and with derivatization

Steroid hormones	Derivatization reagent	LLOQ (pg/mL)		Enhance fold
		Derivatization reagent	Without derivatization reagent	
17αOH-PROG	DMBA	5	5	1
E_1	DMBA	10	5000	500
2-Methoxyestrone	DMBA	20	500	25

Tetrahydrocortisol	DMBA	50	200	4
Corticosterone	DMBA	50	500	10
E_2	DMBA	20	NOFI	-
Pregnandiol	DMBA	50	50	1
19-Hydroxyandrostenedione	DMBA	200	2000	10
Cortol	DMBA	200	500	2.5
11-Desoxycorticosterone	GP	5	5	1
Tetrahydrocorticosterone	GP	5	500	100
5β-Pregnane-3,20-dione	GP	5	50	10
PREG	GP	5	1000	200
E_3	GP	10	20	2
TES	GP	10	100	10
17αOH-PREG	GP	10	NOFI	-
DHEA	GP	20	10000	500
Cortisone	GP	50	50	1
11β,17α,21-Trihydroxy-5β-pregnane-3,20-dione	GP	1000	>40000	>40
PROG	GP	5000	NOFI	-

NOFI: not obtained fragment information

Table S4. Comparison of value of steroid hormone containing carbonyl and hydroxyl with derivatization by DMBA and GP respectively

Substances	Effective value	
	DMBA	GP
2-Methoxyestrone	$3.29e^6$	$6.11e^3$
Tetrahydrocortisol	$1.67e^5$	$1.1e^4$

Corticosterone	$7.74e^{4}$	$9.35e^{3}$
11-Desoxycorticosterone	e^{2}	$4.09e^{3}$
17αOH-PREG	e^{2}	$2.75e^{4}$
Cortisone	$1.04e^{3}$	$2.25e^{5}$
E_3	$3e^{3}$	$2.03e^{5}$
TES	e^{3}	$6.02e^{3}$
